# Somatic Mutations Associated with Aldosterone-Producing Adenomas (APAs)

**DOI:** 10.3390/genes16070778

**Published:** 2025-06-30

**Authors:** Aina Nadheera Abd Rahman, Elena Aisha Azizan

**Affiliations:** Department of Medicine, Faculty of Medicine, Universiti Kebangsaan Malaysia (UKM), Kuala Lumpur 56000, Malaysia

**Keywords:** hypertension, aldosterone-producing adenomas, somatic mutations

## Abstract

Hypertension is a critical health concern as it affects millions of people worldwide and leads to increased risk factors for other diseases such as cardiovascular diseases and stroke. Hypertension is commonly categorized into primary hypertension and secondary hypertension, with the latter frequently curable when caused by the presence of a benign adrenal adenoma that produces excessive adrenal hormones. The incidence rate of these adrenal adenomas is relatively high, in keeping with the hyperplastic/hypermutable characteristic of the adrenal gland. One of the most common functional adrenal adenomas are the aldosterone-producing adenomas (APAs), which develop from the adrenal cortex and, as per the name, produce excessive amounts of the adrenal hormone aldosterone, leading to hypertension. Investigations of genetic causes of these adenomas have revealed that the de novo somatic mutations that commonly cause the increase in aldosterone production mostly involve changes in intracellular concentration. Herein, we review the somatic genetic alterations that have been reported in APAs over the decade.

## 1. Introduction

Hypertension, characterized by elevated blood pressure, is a pervasive condition affecting 1.28 billion adults worldwide, particularly those in low- and middle-income countries [1]. It is often labelled as a “silent killer” as it can quietly progress without overt symptoms to other threatening health conditions such as cardiovascular diseases, renal dysfunction, and stroke [2,3]. According to the World Health Organization (WHO) [1], approximately half of the adults in the world with hypertension are unaware of having the condition. Its insidious nature underscores the challenge of timely detection and intervention. Hypertension can be categorized into either primary or secondary hypertension. Primary hypertension is the most common, accounting for up to 90% of total hypertensives, and has no known cause, thus known as essential or idiopathic hypertension. Secondary hypertension has an identifiable cause, and accounts for around 10% of the cases [4]. Secondary hypertension is commonly caused by a benign adrenal adenoma that produces excess adrenal hormones. These hypertension cases are also commonly called endocrine hypertension.

Examples of hormonally active benign adrenal adenomas include aldosterone-producing adenomas (APAs), cortisol-producing adenomas (CPAs), and phaeochromocytomas (that are not malignant). The APA is the most common form of endocrine hypertension and is thought to originate from the adrenal cortex, the outer layer of the adrenal gland. To note, non-functional adrenal adenomas (or endocrine-inactive adenomas) are actually common and often found “incidentally”, thus sometimes called incidentalomas. It is reported that the frequency of adrenal tumours increases with age, with people aged older than 40 having a prevalence as high as 4–7% [5,6].

Perhaps explaining this high rate of incidence is the finding that the adrenal gland is among the top five organs significantly enriched with non-synonymous mutations [7]. This suggests that adrenal cells have hypermutable characteristics. However, APAs are commonly driven by a well-defined set of recurrent somatic mutations that affect key regulatory genes involved in the excessive aldosterone production phenotype. Interestingly, aldosterone-producing cell clusters (APCCs) containing similar somatic mutations as found in APAs increase with age [8,9,10,11]. Understanding the molecular mechanisms underlying the disease would be of benefit for the management of this common endocrine-active adenoma. Herein, this review summarizes the genes responsible for somatic mutations discovered in APAs over the decade, integrating both well-characterized aldosterone driver genes and more recently reported mutations, including *CADM1* and *SLC30A1*. In addition to outlining the molecular mechanisms, this review also highlights variations of mutation frequency by ethnicity and gender, and the clinical implications.

## 2. Literature Search Strategy

A comprehensive literature search was conducted using PubMed and Scopus databases, covering publications ranging from 2011 until 2024. This time frame was selected to include the emergence of high-throughput sequencing studies and the identification of key driver mutations in APAs following the initial discovery of *KCNJ5* mutations in 2011. Search terms included combinations of Medical Subject Headings (MeSH) and free-text keywords such as “aldosterone-producing adenoma”, “APA”, “primary aldosteronism”, “somatic mutation”, “human”, and names of individual genes reported in the literature, such as *KCNJ5*, *ATP1A1*, *ATP2B3*, *SLC30A1*, *CACNA1D*, *CACNA1H*, *CTNNB1*, *GNA11/Q*, *CADM1*, and *PRKACA.* Included studies comprised original research articles reporting somatic mutations in human APA samples, functional characterization studies, and investigations linking mutation status to clinical phenotypes or outcomes. Excluded materials included case reports lacking frequency data, articles focusing extensively on germline mutations, animal models, or in vitro systems without human validation, and non-English publications. Reference lists of selected articles were also manually screened for additional relevant studies.

## 3. Background

Aldosterone-producing adenomas (APAs) are benign macronodules in the adrenal cortex that autonomously secrete aldosterone and are thus one of the major underlying causes of primary aldosteronism (PA) [12]. The APA is also known as Conn’s syndrome after the first endocrinologist who described the disease in the 1950s, Dr. Jerome Conn [13]. According to a study by Amar et al. [14], it is estimated that between 6 and 13% of hypertensive patients have either APAs (approximately 30% of PA) or another form of PA, bilateral adrenal hyperplasia (BAH). Unilateral APA is a surgically curable form of PA and one of the most common forms of endocrine hypertension [14,15,16,17]. In PA patients, hypertension occurs due to the increased sodium ions in the blood and retention of water by the kidney resulting from the overproduction of aldosterone. It is also important to note the effect of excess aldosterone on cardiovascular function in PA patients when compared to patients with primary hypertension, as aldosterone can stimulate cardiac remodelling and the deposition of vascular collagen [18]. Luckily, this cardiovascular remodelling, such as the increased thickness of carotid intima-media and arterial stiffness, may regress following the adrenalectomy of the culprit lesion [19,20,21]. Several genes are frequently mutated somatically in APAs—*KCNJ5*, *CACNA1D*, *ATP1A1*, *ATP2B3*, and *CTNNB1* [9,10,11,15,22,23,24,25,26,27,28,29,30,31,32,33,34,35,36,37]. More recently, somatic mutations in *GNA11*/*GNAQ* (detected with *CTNNB1* mutations)*, CACNA1H*, *CLCN2*, *CADM1*, *SLC30A1*, and *PRKACA* have also been identified, though rarely, further expanding our understanding of the genetic changes involved in APA development [38,39,40,41,42,43,44,45,46].

One of the best methods of identifying somatic mutations associated with APAs is by using a CYP11B2 (aldosterone synthase) immunohistochemistry (IHC)-guided gene-targeted next-generation sequencing (NGS) approach [36]. This approach provides a more precise and comprehensive genetic analysis compared to conventional hotspot sequencing methods, which only look at specific mutation-prone regions. More importantly, IHC guidance ensures that CYP11B2-expressing cells, which are actively producing aldosterone, are selected for genetic analysis. This is a critical advantage over traditional non-IHC-guided methods, which may inadvertently include non-aldosterone-producing tumour regions or even normal adrenal tissue, which can dilute mutation signals and lead to false-negative results. Additionally, in cases where the tumour cell count or the amount of DNA is low, IHC-guided selection ensures a more targeted approach, enhancing the sensitivity of NGS and improving mutation detection accuracy. This method has been reported to increase the aldosterone-driver mutations in approximately 90% of APAs [8,47,48]. Thus, studies which do not use this method may be under-reporting mutations and misreporting wild-type cases.

## 4. Somatic Mutations Associated with APAs

### 4.1. KCNJ5

**Figure 1:**
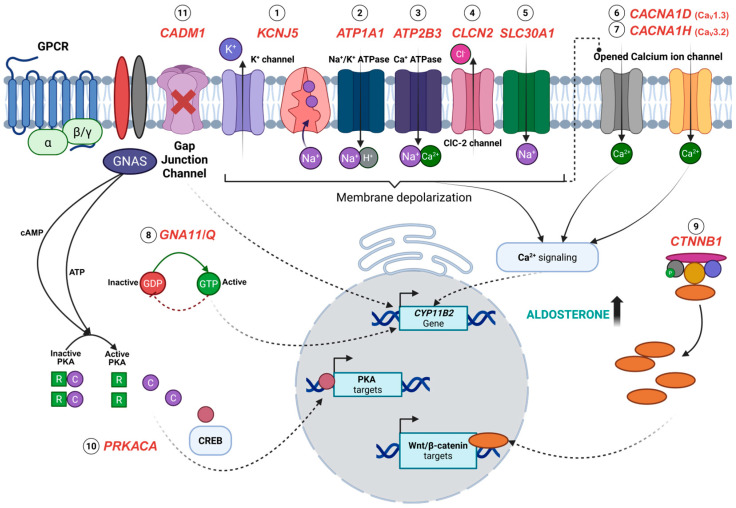
Overview of the pathogenesis of aldosterone-producing adenomas (APAs) associated with somatic mutations *KCNJ5* (1), *ATP1A1* (2), *ATP2B3* (3), *CLCN2* (4), *SLC30A1* (5)*, CACNA1D* (6), *CACNA1H* (7), *GNA11/Q* (8), *CTNNB1* (9), *PRKACA* (10), and *CADM1* (11). Somatic mutations in *KCNJ5*, *ATP1A1*, and *ATP2B3* impair ion transport, resulting in membrane depolarization and elevated calcium concentrations, which are the key drivers of aldosterone synthesis. Similarly, mutations in *CLCN2* and *SLC30A1* alter chloride, zinc, and sodium ion transport, indirectly potentiating calcium signalling. Calcium influx is also enhanced by activating mutations in voltage-gated calcium channels, *CACNA1D* and *CACNA1H*, contributing to the sustained stimulation of *CYP11B2* expression. The constitutive activation of G-protein signalling due to *GNA11* and *GNAQ* mutations, along with aberrant Wnt/β-catenin signalling from *CTNNB1* mutations, leads to an increased transcription of *CYP11B2* and Wnt/β-catenin target genes that promote cellular proliferation and differentiation. Likewise, activating mutations in *PRKACA* enhance the cAMP/PKA pathway activity and the transcription factor cAMP-response element-binding protein (CREB), leading to increased gene expression of PKA targets. Uniquely, *CADM1* mutations disrupt cell–cell communication via gap junctions, which is thought to regulate aldosterone production. Created with BioRender.com.

The gene most frequently mutated with an aldosterone-driver variant in APAs is thought to be the *KCNJ5* gene, encoding for the Voltage-Gated Potassium Channel Subfamily J Member 5. Mechanistically, *KCNJ5* aldosterone-driver mutations result in the loss of potassium selectivity and an increase in sodium influx into the cytoplasm, leading to plasma membrane depolarization (Figure 1) [11]. This subsequently triggers the activation of voltage-gated calcium channels and thus increases intracellular calcium concentration, initiating downstream signaling pathways that lead to elevated aldosterone secretion [49]. The most prevalent aldosterone-driving *KCNJ5* mutations in sporadic APAs are point mutations, particularly p.Gly151Arg and p.Leu168Arg, which in fact account for the majority of reported cases [11,27]. Nonetheless, the pathology of aldosterone production in APA may also be caused by the deletion of amino acids in the *KCNJ5* protein, such as *KCNJ5* p.Ile157_Glu159del and p.Ile157del [23,50]. APAs harbouring *KCNJ5* mutations typically share morphological characteristics with the adrenal cortex zone responsible for cortisol production, known as the zona fasciculata (ZF) [23].

### 4.2. CACNA1D

The other ion channel mutations commonly involved in the pathogenesis of APA are gain-of-function mutations in the *CACNA1D* gene, present in up to 42.7% of APAs. *CACNA1D* encodes the Voltage-Dependent L-Type Calcium Channel Subunit Alpha-1D. Voltage-gated calcium channels (VGCCs) have long been thought to play a key role in stimulating aldosterone production through the influx of calcium ions in zona glomerulosa (ZG) cells when glomerulosa cells are depolarized [51]. In mutant *CACNA1D* associated with APA formations, the continuous activation of the voltage-gated Ca_V_1.3 channel (Figure 1) occurs due to substantial changes to the activation and inactivation of the channel by the mutation [27,34]. APAs harbouring these mutations are thought to have morphological characteristics more similar to the adrenal cortex zone that physiologically produces aldosterone, known as the zona glomerulosa (ZG) [9].

### 4.3. ATP1A1 and ATP2B3

Unlike *CACNA1D*, the *ATP1A1* (encoding the Sodium/Potassium-Transporting ATPase Subunit Alpha-1) aldosterone-driving mutation causes increased intracellular calcium ion concentrations indirectly through the inactivation of pump function, which in turn affects cell membrane potential (Figure 1) [10,52]. The mutations of *ATP1A1* (such as p.Leu104Arg) are also predominantly found in adenomas with cells resembling the ZG (i.e., in ZG-like APAs), as are the *ATP2B3* aldosterone-driving mutations [9]. The *ATP2B3* gene encodes for Plasma Membrane Calcium-Transporting ATPase 3, which is responsible for transporting intracellular calcium ions out of the cells [53]. The aldosterone-driver mutations in *ATP2B3* (such as the p.Leu425_Val426del or p.Val426_Val427del) disrupt the calcium ion binding site, resulting in the accumulation of intracellular calcium, and subsequent stimulation of aldosterone production [28,52,54]. APAs carrying ATPase mutations have been observed to have higher *CYP11B2* mRNA expression levels compared to those with *KCNJ5* mutations, suggesting an increased stimulation of *CYP11B2* mRNA expression in APAs carrying either *ATP1A1* or *ATP2B3* mutations [52]. This finding is corroborated by the high expression of CYP11B2 protein in ATPase-mutant APAs as detected by immunohistochemistry (IHC) staining [55].

### 4.4. CTNNB1

*CTNNB1* (encoding β-catenin) has been found to be mutated in 3–5% of APA patients (Table 1) [33,35]. In Åkerström et al.’s [33] study, all of the mutant APAs had affected the “hotspot” amino acids p.Thr41Ala and p.Ser45Pro. A *CTNNB1* mutation or deletion of exon 3 that encompasses these “hotspot” amino acids, leads to the aberration of Wnt signalling through the inhibition of β-catenin phosphorylation [56]. However, studies have found that Wnt/β-catenin signalling is constitutively active in approximately 70% of APAs, largely due to the downregulation of SFRP2 (secreted frizzled-related protein 2), an endogenous inhibitor of the pathway [57]. Nevertheless, the aldosterone-driver variant is associated with aberrant β-catenin accumulation and is thought to increase the proliferation rate in the adrenal cells, leading to tumour formation [35]. *CTNNB1* mutation can occur independently from the other mutations such as *KCNJ5*, *ATP1A1*, *ATP2B3*, and *CACNA1D*. However, the most overt PA phenotype of patients harbouring a *CTNNB1* aldosterone-driver variant occurs when it is co-present with *GNA11* or *GNAQ* gain of function mutations [46].

### 4.5. GNA11 and GNAQ

Mutations in *GNAQ* and *GNA11*, particularly p.Gln209, are associated with a subset of APAs, impairing GTP hydrolysis and resulting in the persistent activation of downstream signalling cascades that increase aldosterone production [46]. *GNA11* (i.e., p.Gln209Pro and p.Gln209His) and *GNAQ* (i.e., p.Gln209His and p.Gln209Leu) mutations result in the constitutive activation of G-protein subunit alpha q (Gq) and G-protein subunit alpha 11 (Gq11), leading to the activation of downstream signalling pathways that regulate aldosterone synthesis. The activation of these proteins due to mutations is a common feature of tumorigenesis [58], as these G protein α subunits regulate the G protein-coupled receptor (GPCR) [59]. Under normal conditions, Gα subunits cycle between an active GTP-bound state and an inactive GDP-bound state, with GTP hydrolysis terminating the signal. However, somatic mutations in these genes disrupt GTPase activity, leading to prolonged and dysregulated signalling [60]. *GNA11* and *GNAQ* mutations have only been found to co-occur within *CTNNB1* mutant APAs, suggesting a synergistic effect of the mutations on aldosterone production [46]. The double mutations were often found in PA patients presenting during puberty, pregnancy, or menopause. The findings of *GNA11*/*Q* single mutations in hyperplastic regions adjacent to double-mutant APAs indicate that *GNA11*/*Q* mutations may arise prior to *CTNNB1* mutations in the development of these tumours [46]. Functional studies revealed that *GNA11*/*Q* mutations alone were clinically silent unless paired with *CTNNB1* mutations, leading to increased aldosterone secretion and the upregulation of *LHCGR*, the gene encoding for the receptor for luteinizing and pregnancy hormones. This suggests a potential hormonal influence on APA development. A recent case study of a 46-year-old woman with PA further characterized the molecular and histological features of *CTNNB1*-*GNA11* double mutations in APAs, reinforcing the oncogenic synergy between *CTNNB1* and *GNA11*/*Q* mutations in driving aldosterone excess and zona glomerulosa-like characteristics [61]. However, unlike the previous study associating these mutations with hormonal changes, this study suggested that an APA with *CTNNB1*-*GNA11* mutations may develop independently of reproductive hormone fluctuations despite the elevated expression of *LHCGR* (and *GNRHR*) mRNA that is present in the tumour.

### 4.6. CACNA1H

Other than *CACNA1D*, aldosterone-driving mutations in the *CACNA1H* gene similarly affect intracellular calcium levels, as it encodes the alpha-1H subunit of voltage-gated T-type calcium channels (Ca_V_3.2). A gain-of-function germline mutation of variant p.Met1549Val found in the S6 segment of repeat III of Ca_V_3.2 was first identified in 2015 in young-onset PA patients [62]. In 2020, the somatic variant p.Ile1430Thr located in the S5 segment of repeat III was reported in APAs without other known aldosterone-driver mutations [38]. The mutations caused a substitution of amino acids in the Ca_V_3.2 channel, altering its function and leading to dysregulated calcium influx, eventually increasing *CYP11B2* mRNA levels and aldosterone production. Scholl et al. reported that the *CACNA1H* p.Met1549Val variant caused the channel to be more responsive to regular calcium fluctuations, with subtle changes in serum potassium level and angiotensin II signalling essential for maintaining homeostasis, rather than high aldosterone output [62]. A whole-cell patch clamp experiment using mutation-induced HEK293T cells has shown the shift of activation to less depolarizing membrane potentials, leading to a sustained influx of calcium ions at membrane potentials near the resting state, allowing increased calcium entry despite an absence of hyperkalaemia [62]. Although the mechanisms involving the somatic mutation of the *CACNA1H* gene in PA patients are yet to be elucidated, the functional effect of the variant increasing adrenal cell aldosterone production and *CYP11B2* mRNA supports the pathological role of the *CACNA1H* p.Ile1430Thr mutation on the development of primary aldosteronism. Patients with *CACNA1H*-mutated APAs present with common PA features including elevated aldosterone levels, suppressed renin activity, and hypokalaemia [38]. Postoperative follow-up revealed biochemical remission, with patients exhibiting a normalization of serum potassium levels and improvement in blood pressure control [38]. Tumour tissue from these patients showed dense CYP11B2 expression, localized to compact, lipid-poor cells.

### 4.7. CLCN2

The *CLCN2* gene encodes the chloride channel ClC-2, which is responsible for maintaining the ionic balance across cell membranes of intracellular chloride levels, which in turn influence various cellular processes, including hormone synthesis. Initially, *CLCN2* mutations were identified in familial hyperaldosteronism type II (FH-II), where they led to increased chloride permeability, membrane depolarization, and the aberrant activation of voltage-gated calcium channels, ultimately stimulating aldosterone synthesis. Fernandes-Rosa et al. (2018) [39] first reported a de novo p.Gly24Asp germline mutation in *CLCN2* using whole-exome sequencing (WES). Later, Dutta et al. (2019) [40] performed Sanger sequencing and found the same mutation as a somatic alteration in a sporadic aldosterone-producing adenoma (APA), demonstrating its role in both hereditary and tumour-associated forms of PA. The APA with the *CLCN2* mutation was notably small in size (13 mm) and found in a young male patient (i.e., 35 years old) with high plasma aldosterone levels but no other known APA driver mutations [40]. Functional studies using H295R-S2 and HEK293 cells transfected with wild-type and mutant *CLCN2* further confirmed that this mutation abolishes normal voltage-dependent gating of ClC-2, leading to continuous chloride influx and sustained aldosterone overproduction. Scholl et al. (2019) highlighted the importance of these findings, noting that the *CLCN2* mutations in APAs provide a rare example of an anion channel directly contributing to aldosterone dysregulation [63]. Unlike mutations in *KCNJ5* and *CACNA1D*, which disrupt cation homeostasis, *CLCN2* mutations redefine the pathophysiology of PA by linking chloride imbalance to the aldosterone excess. APAs with these mutations may also have a distinct phenotype that is smaller in size and found in younger patients. However, somatic mutations in the *CLCN2* gene are present in only a small percentage of APAs. Rege et al. (2020) identified *CLCN2* somatic mutations in approximately 1.74% of APAs using *CYP11B2*-guided WES and targeted amplicon sequencing [41]. This technique identified a new mutation, c.64-2_74del, in addition to the previously identified c.G71A (p.Gly24Asp) mutation, which also enhanced chloride conductance and led to sustained membrane depolarization.

### 4.8. CADM1

Somatic mutations in the *CADM1* gene have been identified in a subset of APAs contributing to PA. The tumours were generally small and demonstrated strong CYP11B2 staining with histological features consistent with a ZG-like APA phenotype. Postoperative outcomes have included significant improvement in the aldosterone–renin ratio (ARR) and in the two patients reported in the discovery cohort, adrenalectomy resulted in complete clinical cure [43]. *CADM1* encodes a cell adhesion molecule that plays a role in intercellular communication and gap junction integrity [64]. Relatively rare somatic mutations in *CADM1* are reported to have a prevalence of approximately 0.5–1.0% in APAs [43]. This impaired cell–cell signalling leads to dysregulated aldosterone production in mutant APAs [43,65]. These mutations, p.Gly379Asp and p.Val380Asp, are intramembranous variants, typically found in patients with reversible hypertension and periodic PA [43]. Wu et al. (2023) performed a functional experiment using H295R cells transduced with wild-type (WT), mutant, or siRNA specific to *CADM1* [43]. Cells transduced with mutant *CADM1* have been shown to upregulate *CYP11B2* mRNA expression by 10- to 24-fold compared to WT cells, leading to increased aldosterone production. *CADM1* mutations were also found to inhibit gap junction (GJ)-permeable dye transfer, suggesting a role for cell communication in aldosterone regulation [43]. Supportively, the blockade of gap junctions by Gap27 has been shown to increase *CYP11B2* mRNA expression (and aldosterone production) similar to *CADM1* mutations [43].

### 4.9. SLC30A1

The *SLC30A1* gene encodes the zinc efflux transporter ZnT1, the most well-studied member of the ZnT family, which consists of ten transporters. It is widely expressed across various tissues and is the only ZnT protein found in the plasma membrane [42]. *SLC30A1* is part of solute carrier family 30 and plays a crucial role in regulating intracellular zinc levels by exporting zinc from the cytoplasm to the extracellular space or intracellular compartments, thereby protecting cells and tissues from zinc toxicity [66,67,68]. Recent studies utilizing next-generation sequencing (NGS) identified recurrent somatic *SLC30A1* mutations, such as p.Leu49_Leu55del and p.Leu51_Ala57del, in around 0.95–1.89% of PA cases and 1.75–2.94% of APAs in men [42]. These mutations occur close to the zinc-binding site in transmembrane domain II, potentially disrupting zinc transport and affecting ion homeostasis. Functional studies by Rege et al. (2023) suggest that mutant ZnT1 leads to abnormal sodium ion conductance, causing cell membrane depolarization and an increase in cytosolic calcium ion activity [42]. This calcium influx serves as a key trigger for aldosterone production, resulting in its dysregulation. However, the exact mechanisms by which these mutations lead to abnormal sodium conductance remain under investigation.

### 4.10. PRKACA

The *PRKACA* gene encodes the catalytic subunit of PKA, which plays a crucial role in various cellular processes, including hormone synthesis. While *PRKACA* mutations have been reported to occur in approximately 1–2% of APAs, they are more prevalent in another endocrine tumour, cortisol-producing adenomas (CPAs), with studies reporting an occurrence of up to 40% [45,69,70,71,72]. Somatic mutations in *PRKACA*, particularly p.Leu206Arg and the newly identified p.His88Asp (c.262C>G) mutation, have been linked to cAMP/PKA pathway dysregulation, a key driver in endocrine tumour development [73,74]. Typically, PKA activity is regulated by its tetrameric structure, where regulatory subunits (PRKAR1A) keep the catalytic subunits (*PRKACA*) inactive [75]. Upon cAMP binding, the complex dissociates, activating PKA, which then phosphorylates downstream targets like cAMP-response element-binding protein (CREB) and cAMP-responsive modulator (CREM) to regulate gene transcription. The mutation often disrupts the interaction between *PRKACA* and its regulatory subunits, leading to the constitutive activation of PKA and unchecked cAMP signalling, which in turn leads to the upregulation of aldosterone synthase [73]. *PRKACA* mutations, specifically p.Leu206Arg, have been associated with the co-secretion of cortisol in some APAs, leading to the development of Cushing’s syndrome in rare cases [44]. Concurringly, APAs harbouring *PRKACA* mutations often lack CYP11B2 expression, suggesting a limited role for this mutation in aldosterone synthesis but a potential contribution to tumour formation [76,77]. However, the p.His88Asp mutation, identified in exon 4 of *PRKACA*, highlights the potential pathogenic role of PKA dysregulation in APA. His88, positioned at the start of the C-alpha helix, is crucial for maintaining the structural integrity and functional regulation of PKA. It is the only residue in the small lobe of the catalytic core that interacts with Thr198, a critical phosphorylation site necessary for PKA activation. Thus, a mutation at this site could potentially alter PKA activity, leading to disrupted signalling and abnormal cell proliferation, though functional analysis revealed no clear evidence of a gain-of-function effect, suggesting that its role in tumorigenesis may involve alternative mechanisms [44]. Nevertheless, CYP11B2 was strongly expressed in the p.His88Asp-mutated APA.

## 5. Ethnic and Gender Variations in APA Mutations

Population and gender differences in mutation prevalence highlight the importance of genetic and environmental factors that might affect the development of APA in PA patients. A striking observation is the dominance of *KCNJ5* mutations, which are the most prevalent across all populations but exhibit significant variability (Table 1). Among the *KCNJ5*-mutant APAs investigated in several studies, it is found that the mutation mostly affected the East-Asians where it ranged from 53 to 96.7% [25,29,30,31,32,35,37], suggesting a potential genetic predisposition, which might be influenced by evolutionary selection, dietary sodium intake, or differences in APA detection and diagnosis. Meanwhile, in European cohorts, the prevalence is lower, ranging between 33.9 and 46.5% [11,12,22,23,26,28,33,34], whereas among African patients, *KCNJ5* mutations are estimated to occur in 34.2% of cases [36] (Table 1). The lower frequency in Europeans and Africans might indicate the presence of alternative molecular drivers of APA pathogenesis in these populations.

The second most common mutation, *CACNA1D*, follows a markedly different pattern. The overall prevalence of the *CACNA1D* mutation is estimated to be around 10% of APA cases [9,15,27,28,32,34,36,37,78,79]. However, in African patients, *CACNA1D* mutations are found in 42.7% of cases [27,36], making them the predominant genetic alteration among Blacks, and with a frequency significantly higher than in Europeans (8.4%) [15,27,28,34] and East-Asians (5.8%) [32,37] (Table 1). This indicates that *CACNA1D*-driven APAs might be particularly relevant in populations of African descent, potentially due to genetic variations in calcium channel regulation or differential susceptibility to APA formation. The high prevalence in African patients is intriguing, as it could reflect an adaptive evolutionary mechanism related to ion channel function or environmental pressures such as dietary differences. To note, individuals of African ancestry are known to have a higher risk for low-renin hypertension [80].

*ATP1A1* and *ATP2B3* mutations, while generally less frequent than *KCNJ5* and *CACNA1D*, display a distinct distribution pattern. Similar to *CACNA1D* mutant APAs, the prevalence of *ATP1A1* mutations was found to be the highest among the African APA patients (8%) (Table 1), suggesting a potential role in APA pathogenesis across different ethnicities but at a much lower rate [36]. Interestingly, *ATP2B3* mutations are reported in around 2% of both Europeans and East-Asians [15,28,31,32], with a slightly higher prevalence (4.1%) in Africans [36] (Table 1). The relatively low frequency of these mutations overall suggests that they contribute to a more specific subset of APA cases rather than being a major driver of tumour formation.

A unique case is *CTNNB1* mutations, which are strongly associated with Wnt signalling dysregulation in APAs. These mutations are found in 5.1% of Europeans and 3.6% of East-Asians, but notably, they have not been detected in African patients [33,35] (Table 1). This raises questions about whether the Wnt/B-catenin pathway plays a less prominent role in APA formation in populations of African ancestry or whether differences in tumour microenvironment and genetic backgrounds influence the occurrence of these mutations. It is also possible that African cohorts included in these studies were relatively small, limiting the ability to detect rare mutations. Similarly, other somatic mutations including *CACNA1H*, *CLCN2*, *SLC30A1*, *CADM1*, *PRKACA*, and *GNA11*/*Q* are too rare to make claims of ethnic or geographic variations. Nevertheless, some of these rare mutations are involved in broader endocrine and metabolic pathways, suggesting that APA formation can have genetically overlapping processes.

Gender-wise, *KCNJ5* mutations are predominantly found in females, and are found in 56% (18–95%) of female patients but only 30% (1–68%) of males [11,12,22,23,25,26,28,29,30,31,32,33,34,35,36,37] (Table 2). This finding has been observed across multiple studies, corroborating the idea that *KCNJ5*-mutant APAs have gender bias, more frequently occurring in females [11,12,22,23,25,28,29,30,32,33,35,36,37] (Table 2). The reasons behind this bias remain unclear but could involve sex hormones, differences in adrenal cortex physiology, or genetic factors that influence ion channel expression and function. For example, oestrogen has been suggested to modulate potassium channel activity, which thus could contribute to the gender bias observed with aldosterone-driving *KCNJ5* mutations [81].

In contrast, *CACNA1D* mutations are more common in males, with 19% of men carrying the mutation compared to only 8% of women [15,27,28,32,34,36,37] (Table 2). This suggests a potential gender-specific regulatory mechanism in calcium channel-driven APA formation, possibly related to androgen signalling or differences in calcium homeostasis between males and females. Similarly, *ATP1A1* and *ATP2B3* mutations are also more frequently found in males, with *ATP1A1* mutations occurring in 11% of men compared to only 2% of women [15,28,31,32,34,36,37], and *ATP2B3* mutations appearing in 3% of males and just 2% of females [15,28,31,32,36,37] (Table 2). These findings reinforce the idea that these mutations may drive a distinct subset of APA cases, potentially linked to male-specific endocrine regulation. Given that androgen receptors are expressed in the adrenal gland [82], testosterone or other male-specific hormonal influences may modulate APA development in these cases.

Interestingly, *CTNNB1* mutations show the opposite trend, being more common in females (67% vs. 33% in males) [33,35] (Table 2). Given the role of the Wnt/β-catenin pathway in adrenal tumorigenesis, it is possible that hormonal factors, particularly oestrogen, may contribute to the higher prevalence in women. This is supported by findings in adrenocortical carcinomas where Wnt signalling activation is more frequently observed in female patients [83].

**Table 2:** Distribution of somatic mutations between males and females in APAs.

Gene	Male	Female	Sample Size, *n*	References
***KCNJ5* mutations**	**357 (30%)**	**656 (56%)**	**1180**	
p.Gly151Arg; p.Leu168Arg	1 (1%)	7 (88%)	8	[11]
p.Gly151Arg; p.Leu168Arg	31 (24%)	97 (74%)	128	[12]
p.Glu145Gln; p.Gly151Arg; p.Leu168Arg	24 (8%)	112 (39%)	287	[22]
p.Gly151Arg; p.Leu168Arg; p.Ile157del	1 (12.5%)	3 (37.5%)	8	[23]
p.Gly151Arg; p.Leu168Arg	7 (47%)	8 (53%)	15	[25]
p.Gly151Arg; p.Leu168Arg	5 (50%)	5 (50%)	10	[26]
p.Trp126Arg; p.Gly151Arg; p.Thr158Ala; p.Leu168Arg	21 (28%)	53 (72%)	74	[28]
p.Arg115Trp; p.Glu145Gln; p.Gly151Arg; p.Leu168Arg; p.Glu246Gly	9 (35%)	17 (65%)	26	[29]
p.Glu145Gln; p.Gly151Arg; p.Ile157del; p.Thr158Ala; p.Leu168Arg	25 (33%)	50 (67%)	75	[30]
p.Gly151Arg; p.Ile157del; p.Thr158Ala; p.Leu168Arg	62 (68%)	26 (28.6%)	91	[31]
p.Thr148_Thr149insArg; p.Gly151Arg; p.Thr158Ala; p.Leu168Arg	56 (43%)	73 (57%)	129	[32]
NA	16 (17%)	76 (83%)	92	[33]
p.Glu145Lys; p.Gly151Arg; p.Leu168Arg	5 (18%)	5 (18%)	28	[34]
p.Gly151Arg; p.Ile157del; p.Thr158Ala; p.Leu168Arg	48 (41.4%)	68 (58.6%)	116	[35]
p.[Thr148Ile;Thr149Ser]; p.Thr149delinsThrThr; p.Thr149delinsMetAla; p.Gly151Arg; p.Leu168Arg	5 (13%)	20 (57%)	25	[36]
p.Glu145Gln; p.Gly151Arg; p.Thr158Ala; p.Leu168Arg	41 (60%)	36 (95%)	68	[37]
***CACNA1D* mutations**	**63 (19%)**	**25 (8%)**	**331**	
pVal401Leu; p.Gly403Arg; p.Phe747Leu; p.Val1353Met	3 (60%)	2 (40%)	5	[15]
p.Gly403Arg; p.Ile770Met	3 (7%)	2 (5%)	43	[27]
p.Val259Asp; p.Gly403Arg; p.Ser652Leu; p.Leu655Pro; p.Tyr741Cys; p.Phe747Val; p.Phe747Leu; p.Ile750Met; p.Ile750Phe; p.Val979Asp; p.Val981Asn; p.Ala998Ile; p.Ala998Val; p.Val1151Phe; p.Ile1152Asn; p.Pro1336Arg; p.Val1338Met; p.Met1354Ile	18 (67%)	9 (33%)	27	[28]
p.Gly403Arg	0	1 (0.8%)	129	[32]
p.Gly403Arg; p.Phe747Leu; p.Arg990His; p.Val1153Gly	4 (14%)	0	28	[34]
p.Val309Ala; p.Val401Leu; p.Gly403Arg; p.Arg619Pro; p.Ser652Leu; p.Phe747Val/Leu/Cys; p.Ile750Phe/Met; p.Arg990Gly; p.Arg993Thr; p.Ala998Val; p.Cys1007Arg; p.Ile1015Ser; p.Val1151Phe	21 (55%)	10 (29%)	31	[36]
p.Gly403Arg; p.Ser652Leu; p.Phe747Val; p.Ser969Leu; p.Arg990His; p.Ala998Val/Ile; p.Ile1015Thr; p.Val1338Met	14 (21%)	1 (3%)	68	[37]
***ATP1A1* mutations**	**38 (11%)**	**5 (2%)**	**341**	
p.Met102_Leu103del; p.Met102_Ile106del; p.Leu103_Leu104del; p.Leu104Arg; p.Phe956_Glu961del; p.Phe959_Glu961del; p.Glu960_Leu964del	8 (80%)	2 (20%)	10	[15]
p.Gly99Arg; p.Phe100_Leu104del; p.Leu104Arg; p.Val332Gly	8 (89%)	1 (11%)	9	[28]
p.Leu104Arg	2 (2.2%)	0	91	[31]
p.Met102_Leu103del; p.Leu104Arg	4 (3%)	0	129	[32]
p.Phe100_Leu104del; p.Leu104Arg	6 (21%)	1 (4%)	28	[34]
p.Leu104Arg; p.Ile955_Glu960delinsLys	5 (13%)	1 (3%)	6	[36]
p.Leu104Arg; p.Phe959_Glu961delinsLeu; p.Glu960_Ala965delinsAlaLeuVal	5 (7%)	0	68	[37]
***ATP2B3* mutations**	**10 (3%)**	**7 (2%)**	**299**	
p.Thr423_Leu425del; p.Val424_Leu425del; p.Val424_Val426del; p.Val426_Val429del	2 (40%)	3 (60%)	5	[15]
p.Leu424_Val425del; p.Leu425_Val426del; p.Val426_Val427del	1 (33%)	2 (67%)	3	[28]
p.Tyr410Asp	0	1 (1%)	91	[31]
p.Val422-Val426delinsSerThrLeu	1 (1%)	0	129	[32]
p.Val424_Leu425del	2 (5%)	1 (3%)	3	[36]
p.Val424_Leu425del; p.Leu425_Val426del	4 (6%)	0	68	[37]
***CTNNB1* mutations**	**6 (33%)**	**12 (67%)**	**18**	
p.Thr41Ala; p.Ser45Phe; p.Ser45Pro	4 (40%)	6 (60%)	10	[33]
p.Ser45Phe; p.Ser45Pro	2 (25%)	6 (75%)	8	[35]
***GNA11* mutations (co-occurring with *CTNNB1*)**	**1 (4%)**	**10 (37%)**	**27**	
p.Gln209Pro with p.Gly34Arg/p.Ser45Phe/p.Ser45Pro; p.Gln209His with p.Ser33Cys/p.Thr41Ala/p.Ser45Phe	1 (4%)	10 (37%)	27	[46]
***GNAQ* mutations (co-occurring with *CTNNB1*)**	**0**	**5 (18%)**	**27**	
p.Gln209His with p.Gly34Glu; p.Gln209Leu with p.Gly34Arg	0	5 (18%)	27	[46]
***CACNA1H* mutations**	**2 (3%)**	**1 (1%)**	**75**	
p.Ile1430Thr	2 (3%)	1 (1%)	75	[38]
***CLCN2* mutations**	**2 (1%)**	**2 (1%)**	**207**	
p.Gly24Asp	0	1 (8.3%)	12	[39]
p.Gly24Asp	1 (1.3%)	0	80	[40]
p.Gly24Asp; c.64-2_74del	1 (0.9%)	1 (0.9%)	115	[41]
***CADM1* mutations**	**4 (10%)**	**2 (5%)**	**40**	
p.Gly379Asp; p.Val380Asp	4 (10%)	2 (5%)	40	[43]
***SLC30A1* mutations**	**5 (3%)**	**0**	**186**	
p.Leu49_Leu55del; p.Leu51_Ala57del	5 (3%)	0	186	[42]
***PRKACA* mutations**	**0**	**3 (2.1%)**	**142**	
p.His88Asp; p.Leu206Arg	0	2 (2%)	122	[44]
p.Leu206Arg	0	1 (5%)	20	[45]

## 6. Clinical Implications of the Somatic Mutations

The identification of somatic mutations offers significant clinical benefits, particularly for the more common aldosterone-driving mutations, in refining diagnosis, guiding treatment decisions, predicting patient outcomes, and understanding long-term prognoses. Mutation profiling enhances the diagnostic accuracy of APAs, allowing for their differentiation from bilateral adrenal hyperplasia (BAH), and this is important as misclassification can lead to suboptimal treatment strategies [84]. While MRAs are effective in managing hypertension and reducing aldosterone-mediated cardiovascular damage compared to adrenalectomy, incomplete adherence or treatment resistance can result in worsening hypertension, renal dysfunction, and increased cardiovascular events. This underscores the need for an accurate diagnosis of lateralization and the potential for alternative therapeutic targets tailored to specific mutations.

Several studies have found that patients with *KCNJ5*-mutated tumours are typically younger, have a lower potassium level, a higher aldosterone level, and larger adenomas [23,85,86]. The patients with the *KCNJ5* mutation are also reported to have thicker aortic wall but less abdominal aortic calcification when compared to the non-carriers [19]. Interestingly, patients with *KCNJ5*-mutated APAs tend to show significant improvement following adrenalectomy, with many experiencing a complete resolution of hypertension and aldosterone excess postoperatively [87,88]. To note, the progression of the wall thickness was also reported to be reduced by adrenalectomy [19]. Interestingly, patients harbouring a *KCNJ5*-mutant APA have been found to have increased hybrid steroids 18-hydroxycortisol and 18-oxocortisol compared to wild-type patients, most likely due to the ZF cell morphology of the APA [23,30,55,89,90,91,92]. Thus, the production of hybrid steroids in APAs can be a diagnostic marker for *KCNJ5*-mutant APA to identify patients for adrenalectomy. However, there have been case reports of PA recurrence in patients with *KCNJ5*-mutant APAs, thus careful post-adrenalectomy follow-up is recommended to monitor for potential recurrence and ensure optimal long-term management [93]. Interestingly, a series of macrolide antibiotics, including roxithromycin, potently inhibited KCNJ5 mutant channels but not KCNJ5 wild-type channels [94]. Thus, macrolide-derived selective *KCNJ5* mutant inhibitors have the potential to advance the diagnosis and treatment of APAs harbouring *KCNJ5* mutations.

On the other hand, the prognosis for patients with *ATP1A1*, *ATP2B3*, or *CACNA1D* mutations is more complex. While unilateral surgery may still be beneficial, postoperative hypertension has been reported to persist [95,96,97,98], suggesting that these mutations may contribute to asymmetrical bilateral aldosterone excess. Unlike *KCNJ5,* where mutations are commonly found in solitary unilateral APAs, *CACNA1D* mutations along with *ATP1A1* and *ATP2B3* mutations are commonly found in APMs (aldosterone-producing micronodules), which may influence the difference in clinical presentations and prognosis [8,99,100]. Thus, for these patients, long-term pharmacological therapy with mineralocorticoid receptor antagonists (MRAs), such as spironolactone or eplerenone, may remain essential for blood pressure control and cardiovascular protection. Clinical characteristics of patients presenting with ATPase mutations such as blood pressure, tumour size, and serum sodium concentration were similar when compared with the other mutated groups [10]. However, the study that first reported these mutations did find higher preoperative aldosterone levels and lower serum potassium levels in individuals with ATPase-mutant adenomas compared to wild-type adenomas [10]. On the other hand, patients with an APA harbouring a *CACNA1D* mutation were associated with an older age at tumour presentation and smaller tumour size, and did not show female overrepresentation as compared to the *KCNJ5* mutant carriers (Table 2) [9,27,101]. To note, in the study by Scholl et al. [27], the response of normalized blood pressure and resolved biventricular hypertrophy of one patient following treatment with a calcium channel blocker supports specific calcium channel blocker treatment for individuals with *CACNA1D*-mutant APAs.

Somatic mutations in *GNA11*/*Q* and *CTNNB1*, when co-occurring in APAs, appear to demonstrate gender-specific and hormonally influenced clinical patterns. These double mutations have been reported with higher frequency in female patients, particularly in cohorts from the UK and Ireland, where clinical manifestation often occurred during periods of hormonal fluctuations (of LH and HCG) such as during puberty, pregnancy, or menopause [46]. These observations highlight the potential role of gender specific hormones in modulating the phenotypic expression of certain APA genotypes and underline the importance of considering a PA diagnosis in the clinical evaluation and management of female patients presenting with hypertension in the first trimester of pregnancy (when HCG is the highest), as most pregnancy-associated hypertension arises in later trimesters. However, a case report of a Japanese female patient with the same double mutations (*GNA11*/*Q* and *CTNNB1*) presented with primary aldosteronism and hypokalaemia despite having a normal menstrual cycle and in the absence of pregnancy-induced hypertension [61]. Thus, further studies to confirm the LH responsiveness of the double mutant APA are needed. Nevertheless, post-adrenalectomy, the patient achieved clinical remission [61] similar to the cohorts from the UK and Ireland [46], suggesting that double mutant patients are good candidates for adrenalectomy treatment.

## 7. Conclusions

In conclusion, most of the recurrent somatic mutations found in APAs affect the regulation of intracellular calcium ions and have different ethnicity distributions and gender biases. However, these differences and biases could be skewed by insufficient gender data for specific somatic mutations or relatively fewer studies performed in non-European descent cohorts. Thus, further studies are needed to confirm whether these findings are true and if true, to investigate how genetic or environmental factors may lead to these differences and biases. Such insights could ultimately support more personalized management of primary aldosteronism whether through mutation-specific medical therapy or genotype-specific screening strategies.

## Figures and Tables

**Table 1:** Frequency of somatic mutations in aldosterone-producing adenomas (APAs).

Gene	Frequency in Europeans	Frequency in East-Asians	Frequency in Africans	References
***KCNJ5* mutations**	**590/1486 (39.7%)**	**526/784 (67.1%)**	**25/73 (34.2%)**	
p.Gly151Arg; p.Leu168Arg	8/22 (36.4%)	-	-	[11]
p.Gly151Arg; p.Leu168Arg	129/380 (33.9%)	-	-	[12]
p.Glu145Gln; p.Gly151Arg; p.Leu168Arg	157/348 (45.1%)	-	-	[22]
p.Gly151Arg; p.Leu168Arg; p.Ile157del	4/8 (50.0%)	-	-	[23]
p.Gly151Arg; p.Leu168Arg	-	15/23 (65.2%)	-	[25]
p.Gly151Arg; p.Leu168Arg	10/28 (35.7%)	-	-	[26]
p.Trp126Arg; p.Gly151Arg; p.Thr158Ala; p.Leu168Arg	180/474 (38.0%)	-	-	[28]
p.Arg115Trp; p.Glu145Gln; p.Gly151Arg; p.Leu168Arg; p.Glu246Gly	-	26/69 (42.0%)	-	[29]
p.Glu145Gln; p.Gly151Arg; p.Ile157del; p.Thr158Ala; p.Leu168Arg	-	75/108 (69.4%)	-	[30]
p.Gly151Arg; p.Ile157del; p.Thr158Ala; p.Leu168Arg	-	88/91 (96.7%)	-	[31]
p.Thr148_Thr149insArg; p.Gly151Arg; p.Thr158Ala; p.Leu168Arg	-	129/168 (76.8%)	-	[32]
NA	92/198 (46.5%)	-	-	[33]
p.Glu145Lys; p.Gly151Arg; p.Leu168Arg	10/28 (35.7%)	-	-	[34]
p.Gly151Arg; p.Ile157del; p.Thr158Ala; p.Leu168Arg	-	116/219 (53.0%)	-	[35]
p.[Thr148Ile;Thr149Ser]; p.Thr149delinsThrThr; p.Thr149delinsMetAla; p.Gly151Arg; p.Leu168Arg	-	-	25/73 (34.2%)	[36]
p.Glu145Gln; p.Gly151Arg; p.Thr158Ala; p.Leu168Arg	-	77/106 (72.6%)	-	[37]
***CACNA1D* mutations**	**57/682 (8.4%)**	**16/274 (5.8%)**	**32/75 (42.7%)**	
pVal401Leu; p.Gly403Arg; p.Phe747Leu; p.Val1353Met	5/165 (3.0%)	-	-	[15]
p.Gly403Arg; p.Ile770Met	4/15 (26.7%)	-	1/2 (50.0%)	[27]
p.Val259Asp; p.Gly403Arg; p.Ser652Leu; p.Leu655Pro; p.Tyr741Cys; p.Phe747Val; p.Phe747Leu; p.Ile750Met; p.Ile750Phe; p.Val979Asp; p.Val981Asn; p.Ala998Ile; p.Ala998Val; p.Val1151Phe; p.Ile1152Asn; p.Pro1336Arg; p.Val1338Met; p.Met1354Ile	44/474 (9.3%)	-	-	[28]
p.Gly403Arg	-	1/168 (0.6%)	-	[32]
p.Gly403Arg; p.Phe747Leu; p.Arg990His; p.Val1153Gly	4/28 (14.3%)	-	-	[34]
p.Val309Ala; p.Val401Leu; p.Gly403Arg; p.Arg619Pro; p.Ser652Leu; p.Phe747Val/Leu/Cys; p.Ile750Phe/Met; p.Arg990Gly; p.Arg993Thr; p.Ala998Val; p.Cys1007Arg; p.Ile1015Ser; p.Val1151Phe	-	-	31/73 (42.5%)	[36]
p.Gly403Arg; p.Ser652Leu; p.Phe747Val; p.Ser969Leu; p.Arg990His; p.Ala998Val/Ile; p.Ile1015Thr; p.Val1338Met	-	15/106 (14.1%)	-	[37]
***ATP1A1* mutations**	**42/667 (6.3%)**	**11/365 (3.0%)**	**6/73 (8.2%)**	
p.Met102_Leu103del; p.Met102_Ile106del; p.Leu103_Leu104del; p.Leu104Arg; p.Phe956_Glu961del; p.Phe959_Glu961del; p.Glu960_Leu964del	10/165 (6.1%)	-	-	[15]
p.Gly99Arg; p.Phe100_Leu104del; p.Leu104Arg; p.Val332Gly	25/474 (5.3%)	-	-	[28]
p.Leu104Arg	-	2/91 (2.2%)	-	[31]
p.Met102_Leu103del; p.Leu104Arg	-	4/168 (2.4%)	-	[32]
p.Phe100_Leu104del; p.Leu104Arg	7/28 (25.0%)			[34]
p.Leu104Arg; p.Ile955_Glu960delinsLys	-	-	6/73 (8.2%)	[36]
p.Leu104Arg; p.Phe959_Glu961delinsLeu; p.Glu960_Ala965delinsAlaLeuVal	-	5/106 (4.7%)	-	[37]
***ATP2B3* mutations**	**11/639 (1.7%)**	**6/365 (1.6%)**	**3/73 (4.1%)**	
p.Thr423_Leu425del; p.Val424_Leu425del; p.Val424_Val426del; p.Val426_Val429del	5/165 (3.0%)	-	-	[15]
p.Leu424_Val425del; p.Leu425_Val426del; p.Val426_Val427del	8/474 (1.7%)	-	-	[28]
p.Tyr410Asp	-	1/91 (1.1%)	-	[31]
p.Val422-Val426delinsSerThrLeu	-	1/168 (0.6%)	-	[32]
p.Val424_Leu425del	-	-	3/73 (4.1%)	[36]
p.Val424_Leu425del; p.Leu425_Val426del	-	4/106 (3.8%)	-	[37]
***CTNNB1* mutations**	**10/198 (5.1%)**	**8/219 (3.6%)**	**0**	
p.Thr41Ala; p.Ser45Phe; p.Ser45Pro	10/198 (5.1%)	-	-	[33]
p.Ser45Phe; p.Ser45Pro	-	8/219 (3.6%)	-	[35]
***GNA11* mutations (co-occurring with *CTNNB1*)**	**11/27 (40.7%)**	**0**	**0**	
p.Gln209Pro with p.Gly34Arg/p.Ser45Phe/p.Ser45Pro; p.Gln209His with p.Ser33Cys/p.Thr41Ala/p.Ser45Phe	11/27 (40.7%)	-	-	[46]
***GNAQ* mutations (co-occurring with *CTNNB1*)**	**5/27 (18.5%)**	**0**	**0**	
p.Gln209His with p.Gly34Glu; p.Gln209Leu with p.Gly34Arg	5/27 (18.5%)	-	-	[46]
***CACNA1H* mutations**	**8/115 (7.0%)**	**0**	**0**	
p.Ile1430Thr	3/75 (4.0%)	-	-	[38]
***CLCN2* mutations**	**4/207 (1.9%)**	**0**	**0**	
p.Gly24Asp	1/12	-	-	[39]
p.Gly24Asp	1/80	-	-	[40]
p.Gly24Asp; c.64-2_74del	2/115	-	-	[41]
***CADM1* mutations**	**6/40 (15%)**	**0**	**0**	
p.Gly379Asp; p.Val380Asp	6/40 (15%)	-	-	[43]
***SLC30A1* mutations**	**3/118 (2.5%)**	**2/68 (2.9%)**	**0**	
p.Leu49_Leu55del; p.Leu51_Ala57del	3/118 (2.5%)	2/68 (2.9%)	-	[42]
***PRKACA* mutations**	**1/122 (0.8%)**	**1/20 (5%)**	**1/122 (0.8%)**	
p.His88Asp; p.Leu206Arg	1/122 (0.8%)	-	1/122 (0.8%)	[44]
p.Leu206Arg	-	1/20 (5%)	-	[45]
